# Association between infection with *Campylobacter* species, poor oral health and environmental risk factors on esophageal cancer: a hospital-based case–control study in Thailand

**DOI:** 10.1186/s40001-021-00561-3

**Published:** 2021-07-31

**Authors:** Arisara Poosari, Thitima Nutravong, Prakasit Sa-ngiamwibool, Wises Namwat, Supaporn Chatrchaiwiwatana, Piti Ungareewittaya

**Affiliations:** 1 Department of Microbiology, Faculty of Medicine, Khon Kaen University, Khon Kaen, 40002 Thailand; 2 Department of Pathology, Faculty of Medicine, Khon Kaen Universtity, Khon Kaen, 40002 Thailand; 3 Department of Oral Biology, Faculty of Dentistry, Khon Kaen University, Khon Kaen, 40002 Thailand

**Keywords:** *Campylobacter* species, Poor oral hygiene, Risk factor, Esophageal cancer

## Abstract

**Background:**

Previous studies have shown the association between *Campylobacter* species infection and that environmental factors, poor oral hygiene in particular, are linked to an increased risk of esophageal cancer (EC). However, no study has reported on these factors in Thailand. Thus, this study’s objective was to evaluate the impact of the relationship between *Campylobacter* infection and environmental factors on EC incidence in the population of Thailand.

**Methods:**

Data from a case–control study were collected from 105 newly diagnosed EC cases and 105 controls recruited from 2007 to 2017. Infection with *Campylobacter* spp. was detected in the formalin-fixed paraffin-embedded (FFPE) tissue of EC taken from gastroesophageal biopsy specimens obtained from the participants, and evaluated using TaqMan^®^ real-time PCR. Multivariable logistic regression was performed to calculate the odds ratios (ORs) and perform data analysis.

**Results:**

Smoking, alcohol use, a family history of cancer, history of gastroesophageal reflux disease, poor oral hygiene and *Campylobacter* spp. infection were shown to be significant risk factors for EC (*p*  <  0.05). The combination of poor oral hygiene and infection with *Campylobacter* spp. constituted significant risk for EC (*p*  <  0.001). In addition, the risk of EC in subjects co-infected with *C. rectus* and *C. concisus* that practiced poor oral hygiene was even higher and was significant (ORadj  =  4.7; 95% CI 2.41–9.98; *p*  =  0.003).

**Conclusions:**

In Thailand, the major risk factors for EC are smoking status, alcohol drinking, family history of cancer, GERD, poor oral hygiene and *Campylobacter* spp. infection. This study found *Campylobacter* spp. prevalence to be associated with EC and appears to be enhanced by poor oral hygiene, suggesting that a combination of poor oral hygiene and *Campylobacter* species infection may together act as an important etiological risk factor for EC.

**Supplementary Information:**

The online version contains supplementary material available at 10.1186/s40001-021-00561-3.

## Introduction

Esophageal cancer (EC), which primarily affects the upper gastrointestinal tract, is the seventh most common cancer worldwide and a leading cause of death; it was responsible for an estimated 5,08,585 cancer deaths (5% of all cancer deaths) in 2018 [1]. An increase in new cases is reported annually [2]. The etiology of EC is multifactorial and involves various factors, such as the host, environment and infectious agents [3–5]. Previous studies have reported that key environmental risk factors for EC incidence include smoking, alcohol consumption, gastroesophageal reflux disease (GERD), a family history of cancer, poor oral hygiene and *Campylobacter* spp. infection, but the causes of EC vary from country to country [6–15]. Numerous studies have found that cigarette smoking and alcohol consumption are significant risk factors for EC (50 times greater than that of individuals who neither drink nor smoke) [16, 17]. Epidemiological evidence has shown that bacterial infections are associated with cancer and EC etiology [18–20]. Recently, researchers established that *Campylobacter* spp., characterized by oral upper digestive tract microbiota, is a major cause of foodborne bacterial infections in developed and developing countries worldwide [21, 22]. *Campylobacter* spp., highly motile, spiral-shaped Gram-negative bacteria, are increasingly being identified as an upper gastrointestinal tract pathogen and subsequent premalignant lesions of the esophagus and prolonged diarrhea, having previously been primarily associated with colonization of the digestive system [23, 24]. The epidemiology of symptomatic *Campylobacter* spp. infection in humans reflects the complexity and variability of *Campylobacter* sources. Common and major causes of *Campylobacter* infection include the consumption of undercooked meat, contaminated drinking water, unpasteurized dairy products and direct contact with farm animals [25–27]. Modern researchers have focused on characterizing bacterial communities and identifying differences in healthy and diseased states globally [28]. Various studies have revealed that infection with Gram-negative bacteria in the presence of esophageal abnormalities, such as Barrett’s esophagus, plays a vital role in the pathogenesis of inflammation and metaplasia [29, 30]. Recently, significant microbial diversity was demonstrated in esophageal squamous cell carcinoma (ESCC), esophagogastric junction (EGJ) cancer and post-ESCC groups, compared to the healthy control group, with the most significant differences observed between the ESCC and healthy control group participants, in particular using microbial composition analysis. Thus, monitoring the esophageal microbiota may be a useful method of predicting the recurrence of tumor [31]. In one study, *Campylobacter* was shown to colonize the esophagus of most patients with Barrett’s esophagus and was linked to enteritis, periodontal infection and tumor formation; however, it was not identified in the control group [32]. It has been proposed that infection with *C. concisus* is a risk factor for the development of Barrett’s esophagus (BE), a condition that is needed for the development of EC [33]. By contrast, a reversed trend was reported in one study, with a low rate of *Campylobacter* spp. identified in the control and cancer patients, together with a high prevalence of GERD and BE [34]. Therefore, there is a significant gap in knowledge regarding microbial influences on EC, with insufficient data available in the literature. Only a few studies have prospectively analyzed the impact of upper digestive tract microbiota on EC risk. Therefore, the primary objective of the current case–control study was to determine the prevalence of *Campylobacter* spp. in EC tissue using gastroesophageal biopsy specimens obtained from the participants using real-time PCR. A secondary objective was to assess the impact of the combination of *Campylobacter* spp. infection and environmental factors including oral hygiene on EC incidence in the population of northeastern Thailand.

## Material and methods

### Study design and participants

This study involved a hospital-based case–control study of patients admitted to Srinagarind Hospital in the city of Khon Kaen, Northeast Thailand. Case and control specimens were obtained retrospectively from the Pathology Department at Srinagarind Hospital from 2007 to 2017. One hundred and five patients with EC and 105 healthy controls were recruited in this hospital-based case–control study, approved by the Khon Kaen University Ethics Committee for Human Research (HE621269).

The control group patients were admitted with esophagitis at the same time as the cancer cases. The cases and controls were recruited from Srinagarind Hospital in Khon Kaen province, Northeast Thailand, from 2007 to 2017. Data collection was conducted by trained nurses. The EC cases comprised new patients whose diagnosis was clinically and histologically confirmed according to the International Classification of Diseases for Oncology, 3rd Edition (ICD-O-3) codes: C15.3, C15.4, C15.5, C15.8, C15.9 and C16.0. The histological diagnosis was reviewed in each case and confirmed by two pathologists, and the medical reports were obtained from the Pathology Department.

The control subjects were healthy individuals, confirmed upon physical examination, who underwent clinical and biochemical analysis during the same period that the cases group were recruited. Eligible controls included individuals without a history of gastric or esophageal malignancy. The subjects were randomly sampled from patients undergoing routine endoscopy for investigation of presumed nonmalignant conditions, such as GERD. A criterion was that their esophageal biopsies had to be macroscopically normal in appearance. The participants provided written informed consent to participate in the current study.

Data on the subjects were obtained using an interviewer-based structured questionnaire. Prior to data collection, trained staff served as interviewers to ensure the validity of the questionnaire questions. The demographic information sought included age, gender, height, weight, smoking status, alcohol consumption, a family history of cancer, oral hygiene practices and a history of GERD. Data on cigarette smoking included the age that smoking started, the number of cigarettes smoked per day and smoking duration. A smoker was defined as a current smoker who smoked at least one cigarette per day, having done so for at least 6 months. Alcohol consumption was divided into two groups (drinkers and non-drinkers). Drinking status was defined as drinking alcohol at least once a day for at least 6 months; non-drinkers were those who consumed alcohol less than once a month. Detailed information was obtained on the participants’ oral hygiene practices, for example, the daily frequency with which they brushed their teeth (never, less than once a day, twice or more per day), the type of instrument used to clean their teeth, the regularity of dental visits (never, annually and every 2–5 years) and a previous history of gingivitis or periodontal disease.

### Tissue samples

Formalin-fixed paraffin-embedded (FFPE) samples [*N * =  210; *n * =  105 EC samples (case group) and *n * =  105 normal tissue samples (control group)] were collected retrospectively between 2007 and 2017 and retrieved from paraffin blocks stored at the Department of Pathology, Faculty of Medicine, Khon Kaen University. Esophageal squamous cell carcinoma (ESCC) and esophageal adenocarcinoma samples were categorized in the case group, and normal esophageal tissue samples were assigned to the control group. The samples were initially evaluated by a specialist; thereafter, the diagnoses were confirmed using electronic gastroscopy, a histopathology report and ICD-O-3 guidance.

### Laboratory methods

#### DNA extraction and quality control

DNA was extracted from the FFPE tissue. The FFPE esophageal tissue was cut into 10-µM sections placed in a 1.5 microcentrifuge tube. Eight sections from each sample were used for DNA extraction. Prior to sectioning, the microtomes and accessories were cleaned using 70% ethanol alcohol. The DNA of the FFPE tissue samples was extracted using a commercially available system, DNeasy^®^ Blood & Tissue Kits (Qiagen, Hilden, Germany), according to the manufacturer’s instructions. Briefly, eight sections (10 µM each) from each FFPE tissue specimen were deparaffinized in xylene and 95% ethanol. The deparaffinized tissue was washed twice with 95% ethanol; 1000 μl of ethanol was added and vortexed for 30 s and then centrifuged at 13,000 rpm for 5 min. The tissue pellets were then washed with distilled water and air dried. The recommended protocols for DNA FFPE extraction were followed (Qiagen, DNA FFPE Tissue^®^, Germany). The yields and quality of the DNA isolated during the process were determined using a NanoDrop^®^ ND-1000 Spectrophotometer (Thermo Scientific, Wilmington, USA). The housekeeping gene (β-actin) served as an endogenous control to guarantee the DNA quality and was detected using a specific primer (forward 5′-TGTCACAGTGCAAGCTCACTCAGT-3′, reverse 5′-TCCTGAGGAGAAGTCTGCCGTT-3′), following the protocols described elsewhere [35]. The integrity of the extracted DNA samples was confirmed by amplifying the housekeeping gene (β-actin) using SYBR Green-based real-time polymerase chain reaction (real-time PCR). The DNA samples were stored at − 20 °C for further analysis.

#### *Campylobacter* species detection using a TaqMan^®^ real-time PCR assay

To detect *Campylobacter* infection, a TaqMan^*®*^ real-time PCR detection system, which utilizes fluorogenic-labeled probes, was used to determine the infection agent count [36]. Specific TaqMan® PCR primers and fluorescently labeled probes (Additional file 1: Table S1) were designed using the Oligoware^®^ 1.0 software program (Integrated DNA Technology, Coralville, USA). Thereafter, theoretical testing was conducted on the candidate primers and probes, and sequence comparisons were made using the BLAST database (National Institutes of Health, USA). For each infectious agent tested, the TaqMan^®^ PCR assay was performed (a final volume of 20 μl for the PCR reaction). Each reaction comprised 1×  THUNDERBIRD™ qPCR Mix (Toyobo Co. Ltd., Japan), 10 µl of TaqMan^®^ qPCR Mix, 0.3 µM of each forward and reverse primer, 0.2 µM of TaqMan^®^ probe and 50 ng of DNA template. The amplification, data acquisition and *Campylobacter* genus DNA analysis were carried out using an Applied Biosystems^®^ 7500 flats system.

PCR amplification comprised an initial denaturation cycle at 95 °C for 5 min, followed by 40 amplification cycles at 95 °C for 30 s, annealing at 55 °C for 22 s and final extension at 72 °C for 30 s. The *Campylobacter* spp. cycling conditions were as follows*:* initial denaturation with 5 min at 95 °C, followed by 40 amplification cycles at 95 °C for 30 s, a specific Tm set for each primer for 30 s and final extension at 72 °C for 30 s. DNA was extracted from three strains following sequencing of the 16S rRNA genes and used as the positive control as in Table 1, and molecular distilled water was used as the negative control. Both were utilized in each PCR assay run. The samples and controls were tested in duplicate, and those considered positive were replicated following amplification within a cycle of  <  36.

**Table 1:** Demographic characteristics of the esophageal cancer cases and controls

Characteristics	Cases		Controls		*p* value
	N = 105		N = 105		
Gender					
Female	42	(40.0%)	56	(53.3%)	0.053^a^
Male	63	(60.0%)	49	(46.7%)	
Age (mean ± SD)	59.5 ± 8.7		56.7 ± 13.2		0.012^b^
Drinking status					
Non-drinker	33	(31.4%)	64	(61.0%)	< 0.001^a^
Drinker	72	(68.6%)	41	(39.0%)	
Smoking status					
Non-smoker	45	(48.2%)	72	(68.6%)	< 0.001^a^
Smoker	60	(51.8%)	33	(31.4%)	
Body mass index; BMI (kg/m^2^) (mean ± SD)	24.1 ± 3.62	23.4 ± 3.46			0.490^b^
< 23.00	49	(46.7%)	54	(51.4%)	
≥ 23.00	56	(53.3%)	51	(48.6%)	
Marital status					0.964^a^
Single	9	(8.6%)	9	(8.6%)	
Married	88	(83.8%)	89	(84.8%)	
Separated	8	(7.6%)	7	(6.7%)	
Family history of cancer in first-degree relatives					
No	27	(25.7%)	60	(57.1%)	< 0.001^a^
Yes	78	(74.3%)	45	(42.9%)	
Personal history of gastroesophageal reflux disease					
No	25	(23.8%)	65	(61.9%)	< 0.001^a^
Yes	80	(76.2%)	40	(38.1%)	
Poor oral hygiene					
No	33	(31.4%)	55	(52.4%)	0.002^a^
Yes	72	(68.6%)	50	(47.6%)	
*Campylobacter *genus infection					
Negative	44	(41.9%)	80	(76.2%)	< 0.001^a^
Positive	61	(58.1%)	25	(23.8%)	

^a^*p* value differences between cases and controls were detected using the Chi-squared test

^b^*p *value differences between cases and controls were detected using the Wilcoxon rank-sum test

### Statistical analysis

The demographic characteristics of the subjects were summarized using descriptive statistics. The categorical data were expressed using frequencies and percentages, and the continuous data, such as the age of the subjects, were depicted by mean  ±  standard deviation (SD), median, and minimum and maximum range. Bivariate analysis was performed using simple logistic regression to determine the association between the independent factors and EC, without controlling for confounding variables. The crude odds ratio (OR_crude_) and the 95% confidence interval (95% CI) were also calculated. Multivariable unconditional logistic regression was used to compute adjusted odds ratios (ORadj) and their 95% confidence intervals (95% CI) for the association between EC risk factors and EC, while controlling for the effects of confounding variables. Candidate variables for the multivariable analysis were selected according to two criteria: firstly, variables in the crude analysis which were found to have a *p *value of less than 0.25, and secondly, variables shown from a literature review to have an association with esophageal cancer. The method of backward stepwise elimination was used as the model fitting strategy. A likelihood ratio test was performed to assess the goodness-of-fit of the final model. The test statistics were two-sided, and a *p *value less than 0.05 was considered statistically significant. The statistical analysis was performed using Stata^®^ software (version 13.0).

## Results

### Demographic characteristics

The characteristics of the 105 EC cases and 105 controls are provided in Table 1. The gender and age distribution differed between the cases and controls (men, 60% and 47%, respectively; women, 40 and 53%, respectively). The mean age (±SD) of the men and women was 59.5 years (±  8.7) and 56.7 years (±  13.2), respectively. The demographics of the subjects’ lifestyle habits were evaluated. Drinking status, smoking and poor oral hygiene were more prevalent among the cases than the controls. The frequency of a family history of cancer and a personal history of GERD differed between the cases and controls (*p * <  0.05). *Campylobacter* spp. infection was identified in 61 cases (58%) and 25 controls (24%).

### Risk factors for esophageal cancer

The primary outcomes of the multivariable analysis are depicted in Table 2. Factors found to be significantly associated with EC were alcohol consumption, smoking status, a family history of cancer in a first-degree relative, a personal history of GERD and poor oral hygiene. The dose–response relationship between poor oral hygiene and EC risk was statistically significant (*p*  <  0.05). Poor oral hygiene increased the odds of developing EC by 49% (OR_adj_  =  3.0; 95% CI 1.44–6.26; *p * <  0.001).

**Table 2:** Crude and adjusted odds ratios for the association of esophageal cancer and various risk factors

Variables	Cases		Control		OR_C_ (95% CI)	OR_adj_ (95% CI)	*p* value
	*N * = 105		*N* = 105				
Gender							0.261
Female	42	(40.0%)	56	(53.3%)	1.0 (reference)	1.0 (reference)	
Male	63	(60.0%)	49	(46.7%)	1.7 (0.98–2.96)	1.3 (0.77–2.53)	
Age (years)							0.339
< 60	14	(13.3%)	35	(33.4%)	1.0 (reference)	1.0 (reference)	
≥ 60	91	(86.7%)	70	(66.6%)	1.8 (0.99–3.01)	1.4 (0.71- 2.81)	
Body mass index; BMI (kg/m^2^)							0.912
< 23.00	49	(46.7%)	54	(51.4%)	1.00 (reference)	1.00 (reference)	
≥ 23.00	56	(53.3%)	51	(48.6%)	1.21 (0.71–2.08)	1.03 (0.55–1.95)	
Marital status							0.810
Single	9	(8.6%)	9	(8.6%)	1.00 (reference)	1.00 (reference)	
Married	88	(83.8%)	89	(84.8%)	0.98 (0.37–2.61)	0.01 (0.35–2.93)	
Separated	8	(7.6%)	7	(6.7%)	1.14 (0.28–4.21)	1.11 (0.26–5.63)	
Drinking status							0.016
Non-drinker	33	(31.4%)	64	(61.0%)	1.0 (reference)	1.0 (reference)	
Drinker	72	(68.6%)	41	(39.0%)	3.4 (1.93–6.02)	3.2 (1.25–8.62)	
Smoking status							0.015
Non-smoker	45	(48.2%)	72	(68.6%)	1.0 (reference)	1.0 (reference)	
Smoker	60	(51.8%)	33	(31.4%)	3.3 (1.86–5.77)	3.5 (1.28–9.56)	
Family history of cancer in first-degree relatives							0.001
No	27	(25.7%)	60	(57.1%)	1.0 (reference)	1.0 (reference)	
Yes	78	(74.3%)	45	(42.9%)	3.8 (2.15–6.91)	3.7 (1.69–8.12)	
Personal history of gastro esophagus reflux diseases (GERD)							0.008
No	25	(23.8%)	65	(61.9%)	1.0 (reference)	1.0 (reference)	
Yes	80	(76.2%)	40	(38.1%)	4.2 (2.86–9.44)	2.8 (1.29–5.62)	
Poor oral hygiene							< 0.001
No	33	(31.4%)	55	(52.4%)	1.0 (reference)	1.0 (reference)	
Yes	72	(68.6%)	50	(47.6%)	2.8 (1.36–4.21)	3.0 (1.44–6.26)	

*p* value from unconditional logistic regression

*ORc* crude odds ratio; *ORadj* adjusted odds ratio; *95% CI* 95% confidence interval

#### *Campylobacter* species infection

The results of *Campylobacter* spp. infection relative to EC are detailed in Table 3. *Campylobacter* infection was associated with an increased risk of EC, and this finding was statistically significant (OR_adj_  =  4.8; 95% CI 2.26–10.04; *p * <  0.001). *C. concisus* and *C. rectus* were also significantly associated with EC (*p * <  0.001). In addition, subjects who were co-infected with *C. rectus* and *C. concisus* were at increased odds ratio of EC, and the association between this risk factor and EC was statistically significant (OR_adj_  =  3.4, 95% CI 1.34–6.61; *p * =  0.002).

**Table 3:** The relationship between *Campylobacter *species and esophageal cancer risk

Variables	Cases		Controls		OR_C_ (95% CI)	OR_adj_ (95% CI)	*p* value
	*N* = 105		*N* = 105				
Gender							
Female	42	(40.0%)	56	(53.3%)	1.0 (reference)	1.0 (reference)	0.261
Male	63	(60.0%)	49	(46.7%)	1.7 (0.98–2.96)	1.3 (0.77–2.53)	
Age (years)							
< 60	14	(13.3%)	35	(33.4%)	1.0 (reference)	1.0 (reference)	0.339
≥ 60	91	(86.7%)	70	(66.6%)	1.8 (0.99–3.01)	1.4 (0.71–2.81)	
*Campylobacter* species infection							
Negative	44	(41.9%)	80	(76.2%)	1.0 (reference)	1.0 (reference)	< 0.001
Positive	61	(58.1%)	25	(23.8%)	4.4 (2.45–8.03)	4.8 (2.26–10.04)	
Type of *Campylobacter* species infection							
* Campylobacter rectus* infection							
Negative	70	(66.7%)	90	(85.7%)	1.0 (reference)	1.0 (reference)	0.027
Positive	35	(33.3%)	15	(14.3%)	3.3 (2.25–8.37)	2.8 (1.11–6.10)	
* Campylobacter concisus* infection							
Negative	59	(56.2%)	87	(84.8%)	1.0 (reference)	1.0 (reference)	0.004
Positive	46	(43.8%)	18	(15.2%)	3.0 (1.52–5.93)	3.1 (1.42–6.77)	
Co-infection							
Negative	74	(70.4%)	95	90.0%	1.0 (reference)	1.0 (reference)	0.002
Positive	31	(29.6%)	10	(10.0%)	3.8 (1.83–8.63)	3.4 (1.34–6.61)	

Co-infection with *Campylobacter rectus* and *Campylobacter concisus*

*p* values obtained using unconditional logistic regression

*ORc* crude odds ratio; *ORadj* adjusted odd ratio; *95% CI* 95% confidence interval

#### A combination of poor oral hygiene and *Campylobacter* species infection

The impact of the combination of poor oral hygiene and infection with *Campylobacter* spp. as a risk factor for EC was found to be statistically significant at *p*  <  0.001 (Table 4). The association between EC and poor oral hygiene in combination with *C. concisus* infection was significantly greater than that between EC and poor oral hygiene in combination with *C. rectus* (OR_adj_  =  4.2; 95% CI 1.88–9.47; *p * <  0.001; OR_adj_  =  3.1, 95% CI 1.33–7.38; *p * =  0.008, respectively). The risk of EC was slightly higher in subjects who were co-infected with *C. rectus* and *C. concisus* and who practiced poor oral hygiene (an increased OR), and the risk associated with these combined factors was statistically significant (OR_adj_  =  4.7; 95% CI 2.41–9.98; *p * <  0.003).

**Table 4:** The relationship between poor oral hygiene behavior and *Campylobacter species* infection as a risk factor for esophageal cancer

Variables	Cases		Controls		OR_adj_ (95% CI)	*P *value
	*N* = 105		*N* = 105			
*Campylobacter* spp. infection plus poor oral hygiene						
Negative	62	(59.1%)	90	(85.7%)	1.0 (reference)	< 0.001
Positive	43	(40.9%)	15	(14.3%)	4.4 (2.31–9.81)	
Type of *Campylobacter* species infection						
* Campylobacter rectus* infection plus poor oral hygiene						
Negative	81	(77.1%)	96	(91.4%)	1.0 (reference)	0.008
Positive	24	(22.9%)	9	(8.6%)	3.1 (1.33–7.38)	
* Campylobacter concisus *infection plus poor oral hygiene						
Negative	73	(69.5%)	95	(90.4%)	1.0 (reference)	< 0.001
Positive	32	(30.5%)	10	(9.6%)	4.2 (1.88–9.47)	
Co-infection plus poor oral hygiene						
Negative	69	(65.7%)	98	(93.3%)	1.0 (reference)	0.003
Positive	36	(34.2%)	7	(6.7%)	4.7 (2.41–9.98)	

Co-infection with *Campylobacter rectus* and *Campylobacter concisus*

*p* values were obtained using unconditional logistic regression and were adjusted for gender and age

*ORadj* adjusted odds ratio; *95% CI* 95% confidence interval

## Discussion

The objective of the current study was to evaluate the relationship between environmental risk factors, in particular microbial factors, and their association with the incidence of EC. In Thailand, the major risk factors for esophageal cancer appear to be smoking status, alcohol drinking, a family history of cancer in a first-degree relative, a personal history of GERD, poor oral hygiene and *Campylobacter* spp. infection. Our results suggest that, in this multifactorial cancer, *Campylobacter* spp. may well be a risk factor. This study found *Campylobacter* spp. prevalence is associated with EC and seems to be enhanced by poor oral hygiene, suggesting that a combination of poor oral hygiene and *Campylobacter* species infection may together act as an important etiological risk factor for EC. The association between *Campylobacter* spp. and EC is inconclusive based on current findings in the literature, and the prevalence of *Campylobacter* spp. in patients with EC has not yet been reported in Thailand.

Thus, a case–control study was conducted to identify *Campylobacter* spp*.* DNA in the esophageal tissue obtained from patients with EC, and compare it with that of the controls using PCR. The identification of *Campylobacter* spp. was confirmed using TaqMan^®^ real-time PCR. The prevalence of *Campylobacter* spp*.* in the esophageal tissue of 105 cases and 105 controls was 58 and 24%, respectively. In the current study, EC was identified more frequently in men than in women. Similar results have been reported in South Thailand [37]. By contrast, a higher incidence of EC in women, compared to men, has been described in Europe [38]. In the current study, the mean age of the EC patients was 59.5 years, which is similar to that of the EC population (a mean age of 57.8 years) recorded using a cancer registry in Khon Kaen, Thailand [39]. Therefore, it was assumed that the finding in the current research on EC cases was representative of this disease in northeast Thailand. This is one of a few studies that has evaluated the relationship between *Campylobacter* spp. and OC by evaluating esophageal tumor tissue specimens.

The prevalence of *Campylobacter* spp. was assessed in archived FFPE tissue derived from EC cases diagnosed between 2007 and 2017. A large proportion (41%) of *Campylobacter* spp. DNA was identified in the tumor specimens, which demonstrated that *Campylobacter* spp. infection was associated with EC progression, and *Campylobacter* infection increased EC risk, and this finding was statistically significant (OR_adj_  =  4.8; 95% CI 2.26–10.04; *p*  <  0.001).

Similarly, *C. concisus* and *C. rectus* were significantly associated with EC (*p*  <  0.001). In addition, the risk of EC increased in subjects who were co-infected with *C. rectus* and *C. concisus* and this finding had statistical significance (OR_adj_  =  3.4; 95% CI 1.34–6.61; *p*  =  0.002). Specifically, the findings in the current study provided evidence of an association between a combination of poor oral hygiene and *Campylobacter* spp. infection and EC risk. In general, *C. rectus* and *C. concisus* are associated with poor oral health, including periodontal disease. A prior population-based study identified an association between poor oral health-periodontal disease and orodigestive cancer due to mono- or polymicrobial infection, which resulted in an increase in proinflammatory cytokines, such as interleukin (IL)-1β, IL-6, IL-8 and tumor necrosis factor alpha (TNFα), ultimately inducing gene expression relating to cell proliferation, for example, oncogenes [40]. It has been shown that *C. concisus* favors colonization in patients with GERD and Barrett’s esophagus (i.e., an increased bacterial count) accompanied by a significant decrease in bacterial count for all other genera [41]. *Campylobacter* spp. was also identified to a greater extent in the biopsy samples of EC cases compared to those in the control group [42]. *C. concisus* was shown to induce IL-18, TNFα and p53 expression in Barrett’s esophageal cell lines. An in vitro study indicated the relationship between esophageal colonization by *C. concisus* and the epithelial production of TNFα and IL-18, known to induce both an innate and adaptive immune response and to increase CDX1 and COX-2 expression. In turn, this escalates the rate of colonization and chronic inflammation, which has been shown to lead to disease progression in GERD, Barrett’s esophagus and EC [33, 43].

Thus, the findings in the current study provide evidence of a significant association between a combination of poor oral hygiene—*Campylobacter* spp. infection and EC risk. Similarly, smoking status, alcohol use, a family history of cancer in a first-degree relative and a personal history of GERD were associated with EC in our sample; the magnitude of these effects was strong enough to achieve statistical significance. Therefore, our results may have policy implications for population-based education and awareness throughout Thailand; however, it will be necessary to confirm these findings using a larger sample size and an experimental design that facilitates an evaluation of the mechanistic pathways that link oral hygiene and EC before serious consideration can be given to implementing interventions.

Limitations and bias should be taken into consideration. A main limitation concerns the retrospective cross-sectional nature of the case–control design; accordingly, a cause–effect relationship between poor oral health and *Campylobacter* spp. infection and EC risk could be established. Firstly, recall bias is usually a limitation in case–control studies, although using patients as controls may have been beneficial in limiting this bias. The behavior data of risk factors assessment were made based on an interviewer-based structured questionnaire, which probably introduced some recall error in the response of participants. Face-to-face interviews were thus conducted in the presence of kin to help improve the accuracy of their answers. Furthermore, information bias and recall bias were unlikely, because all participants were blind to the study hypothesis. Secondly, the control subjects were selected from patients with esophagitis undergoing routine investigations based on a presumption of non-malignancy and good health. Nonetheless, disease misclassification was possible because some of the control subjects may have subsequently been diagnosed with EC because, typically, it is slow-growing and remains clinically ‘silent’ until the late stages.

This study had three important strengths. Firstly, it was a hospital-based case–control study that recruited 105 patients with newly diagnosed EC (the diagnoses were confirmed following clinical and histological analyses after a review of each sample by two pathologists in the Department of Pathology at the Faculty of Medicine, Khon Kaen University) and 105 healthy controls. Secondly, this is the first published study to describe the relationship between poor oral hygiene–infection with *Campylobacter* spp. and EC in human subjects. In addition, the study demonstrated a dose–response relationship between poor oral hygiene–infection with *Campylobacter* spp. and EC risk, which is sound evidence of an underlying causal relationship. Thirdly, the data collection was conducted by trained nurses, the laboratory that evaluated *Campylobacter* spp. infection used real-time PCR (TaqMan^®^ probes), and unconditional logistic regression was employed for the data analysis.

In conclusion, in this study the factors of alcohol consumption, smoking status, a family history of cancer in a first-degree relative, a personal history of GERD, poor oral hygiene and *Campylobacter* spp. infection were established to be significant risk factors for EC in Thailand. In addition, the prevalence of *Campylobacter* spp. in EC cases was significantly higher than that in the controls. Therefore, a combination of poor oral hygiene and infection with *Campylobacter* spp*.* were a significant risk factor for EC*.* Subjects who were co-infected with both *C. rectus* and *C. concisus* and characterized by poor oral hygiene behavior were at increased risk of esophageal cancer, and this finding had statistical significance, which suggests that a combination of *Campylobacter* spp. infection and poor oral health is a potential and significant risk factor for EC. Hence, further prospective cohort studies and studies that utilize larger sample sizes are warranted to confirm the present study’s findings, and establish the possible mechanisms for *Campylobacter* spp. etiology and virulence gene infection in EC.

## Supplementary Information

**Additional file 1**: **Table S1.** The detection of bacteria using polymerase chain reaction primers and TaqMan^®^ probes.

## Data Availability

The datasets during and/or analyzed during the current study are available from the corresponding author on reasonable request.

## Abbreviations

°C: Celsius
95% CI: 95% Confidence interval
DNA: Deoxyribonucleic acid
OR adj: Adjusted odds ratios
OR c: Crude odds ratios
TaqMan^®^ real-time PCR: Polymerase chain reaction
SD: Standard deviation
FAM: 6‐Carboxyfluorescein-labeled reporter dye
HEX: 7‐Hexachloro-6-carboxy-fluorescein-labeled reporter dye
TAMRA: 6‐Carboxytetramethyl‐rhodamine-labeled quencher dye
