# Supplementary material for: Association between infection with Campylobacter species, poor oral health and environmental risk factors on esophageal cancer: a hospital-based case–control study in Thailand
Source: Eur J Med Res. 2021 Jul 31;26:82. doi: 10.1186/s40001-021-00561-3 (PMC8325836; doi:10.1186/s40001-021-00561-3)
Supplement: Supplementary file 1 — Additional file 1: Table S1. The detection of bacteria using polymerase chain reaction primers and TaqMan® probes. [file 40001_2021_561_MOESM1_ESM.docx]

**Table S1.** The detection of bacteria using polymerase chain reaction primers and TaqMan^®^ probes

| Infection agents  (GenBank accession numbers) | Sequence (5’-3’) | Amplicon size (bp) | References |
| --- | --- | --- | --- |
| ***Campylobacter* species (Cam16s RNA)** | | | |
| Forward | 5’-CTGCTTAACACAAGTTGAGTAGG-3’ | 267 | Lund et al. (44) |
| Reverse | 5’-TTCCTTAGGTACCGTCAGAA-3’ |  |  |
| Probe | FAM-5’-CGCTCCGAAAAGTGTCATCCTCC-TAMRA -3’ |  |  |
| ***Campylobacter rectus* (AF 035193.1)** | | | |
| Forward | 5’-CACCCGATAACCCTACTCCTCCTA-3’ | 132 | Sencimen et al. (45) |
| Reverse | 5’-GATCCGTTCCATCAGTACCCACTA-3’ |  |  |
| Probe | HEX-5’-CCGGTACCGAATCCTGAGGAACCA-TAMRA-3’ |  |  |
| ***Campylobacter concisus* (CP012541.1)** | | | |
| Forward | 5’-GCCTAGCTCTTCACTGATAAC-3’ | 216 | The current study |
| Reverse | 5’-CCCATACTTCATCACAAACCC-3’ |  |  |
| Probe | FAM-5’-TACTTGCTCAAGCACTGGCAATA-TAMRA-3’ |  |  |

Abbreviation: FAM, FAM, 6‐carboxyfluorescein-labelled reporter dye; HEX, 7‐hexachloro-6-carboxy-fluorescein-labelled reporter dye; TAMRA, 6‐carboxytetramethyl‐rhodamine-labelled quencher dye

References

44. Lund, M., Nordentoft, S., Pedersen, K., & Madsen, M. Detection of Campylobacter spp. in chicken fecal samples by real-time PCR. Journal of Clinical Microbiology. 2014; 42(11): 5125–5132.

45. Sencimen, M., Saygun, I., Gulses, A., Bal, V., Acikel, C. H., & Kubar, A. Evaluation of periodontal pathogens of the mandibular third molar pericoronitis by using real time PCR. International Dental Journal. 2014; 64(4): 200–205.
